# Variability of fluorescence intensity distribution measured by flow cytometry is influenced by cell size and cell cycle progression

**DOI:** 10.1038/s41598-023-31990-1

**Published:** 2023-03-25

**Authors:** Radek Fedr, Zuzana Kahounová, Ján Remšík, Michaela Reiterová, Tomáš Kalina, Karel Souček

**Affiliations:** 1 Department of Cytokinetics, Institute of Biophysics of the Czech Academy of Sciences, Královopolská 135, 612 00 Brno, Czech Republic; 2 International Clinical Research Center, St. Anne’s University Hospital Brno, Brno, Czech Republic; 3 Human Oncology and Pathogenesis Program, Memorial Sloan Kettering Cancer Center, New York, NY 10065 USA; 4 CLIP - Childhood Leukaemia Investigation Prague, Department of Pediatric Haematology and Oncology, Second Faculty of Medicine, Charles University and University Hospital Motol, Prague, Czech Republic; 5 Department of Experimental Biology, Faculty of Science, Masaryk University, Brno, Czech Republic

**Keywords:** Cell division, Flow cytometry

## Abstract

The distribution of fluorescence signals measured with flow cytometry can be influenced by several factors, including qualitative and quantitative properties of the used fluorochromes, optical properties of the detection system, as well as the variability within the analyzed cell population itself. Most of the single cell samples prepared from in vitrocultures or clinical specimens contain a variable cell cycle component. Cell cycle, together with changes in the cell size, are two of the factors that alter the functional properties of analyzed cells and thus affect the interpretation of obtained results. Here, we describe the association between cell cycle status and cell size, and the variability in the distribution of fluorescence intensity as determined with flow cytometry, at population scale. We show that variability in the distribution of background and specific fluorescence signals is related to the cell cycle state of the selected population, with the 10% low fluorescence signal fraction enriched mainly in cells in their G0/G1 cell cycle phase, and the 10% high fraction containing cells mostly in the G2/M phase. Therefore we advise using caution and additional experimental validation when comparing populations defined by fractions at both ends of fluorescence signal distribution to avoid biases caused by the effect of cell cycle and cell size.

## Introduction

Cell cycle is an essential biological process that significantly contributes to the transcriptional heterogeneity in cell differentiation^1^, cell death^2^, and carcinogenesis^3^. Exploration of data obtained with single-cell RNA sequencing (scRNA-seq) revealed that the cell cycle and cell volume can act as sources of bias, introducing within-cell-type phenotypic and functional heterogeneity^4–7^. Unbiased cell clustering may therefore be obtained by correcting for cell cycle effects^7,8^. Several strategies were developed to remove cell cycle effects from scRNA-seq (for review see^8^) and mass cytometry data^6^. Besides scRNA-seq and mass cytometry, current state-of-the-art fluorescence-based flow cytometry allows measurement of 40+ colours simultaneously^9,10^, and represents a re-emerging technology for large scale single-cell analysis^11^, with deeper understanding the cell cycle and cell volume effects in polychromatic flow cytometry data now becoming more than necessary. Two major technical limitations of flow cytometry are background fluorescence, sometimes referred to as autofluorescence, and spreading error, which can contribute to the incorrectly identified heterogeneity within cell populations^12^. The background, native fluorescence is a normal characteristic of every particle, cell and tissue. Background fluorescence is influenced by cellular phenotype^13,14^, metabolic state^15,16^, and proliferation rate^17^. A number of endogenous fluorophores have been described, including aromatic amino acids, cytokeratines, collagen and elastin, NAD(P)H, flavins, fatty acids, vitamin A derivatives, porphyrins and lipofuscin, and these can be exploited as intrinsic biomarkers^18^. These molecules are excited by and emit over a broad range of wavelengths and often overlap the spectra of commonly used fluorescent probes^19^. This interesting phenomenon, together with the technological advancements, opened a large field of investigation and application of autofluorescence in biological research^14,20,21^ and biomedical diagnosis^22,23^. On the other hand, it must be noted as an obstacle and a potential pitfall of fluorescence-based techniques^12,24^.

Here, we investigated the association between cell cycle status, cell size, and the variability of fluorescence intensity distribution as measured by flow cytometry. We demonstrated that the variability in the distribution of both background and specific fluorescence signal is related to the cell cycle state of the measured cell population. Cells with low fluorescence signal are enriched in smaller cells, mostly in G0/G1 phase, while the cells with high fluorescence signal are larger and in G2/M phase. We argue that the data interpretation from experiments comparing the populations defined as “low” versus “high” in terms of symmetric selection of fractions at both ends of fluorescence signal distribution could be misleading. Investigators should take into account the effect of cell cycle and cell size and corroborate such findings with other techniques.

## Results

### Fluorescence background distribution is related to the cell cycle status in living and fixed cells

**Figure 1:**
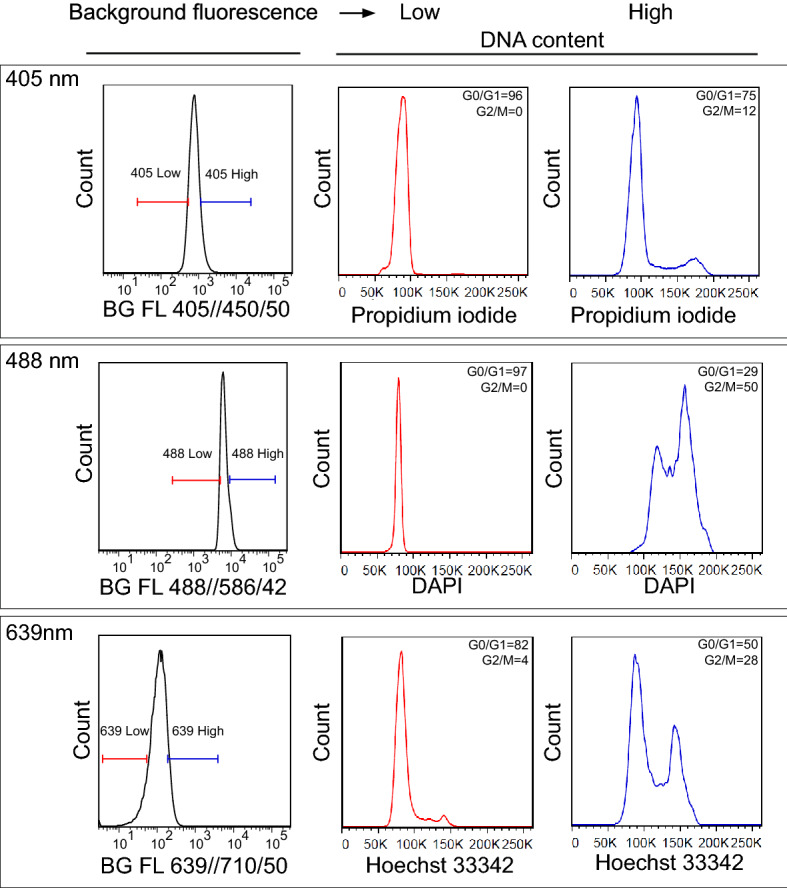
Fluorescence background distribution is associated with cell cycle state in live and fixed cells. HCT 116 cells were stained using a series of DNA dyes, in fixed (propidium iodide, DAPI) or native (Hoechst 33342) conditions. Background fluorescence was analyzed using 405, 488, and 639 nm lasers and an array of detectors (425 up to 810 nm) that were separated from the optical line for the particular DNA dye. Samples were analyzed using flow cytometry (BD FACSAria II SORP). Dead cells were excluded based on LIVE/DEAD staining, fractions of the cells with low (bottom 10%) and high (top 10%) background fluorescence were gated for DNA content (cell cycle) analysis. The values of G0/G1 and G2/M phase represent the proportion of cells. Data are representative from at least three independent repetitions. For total DNA content distribution of the entire population, see Supplementary Fig. 8A.

**Figure 2:**
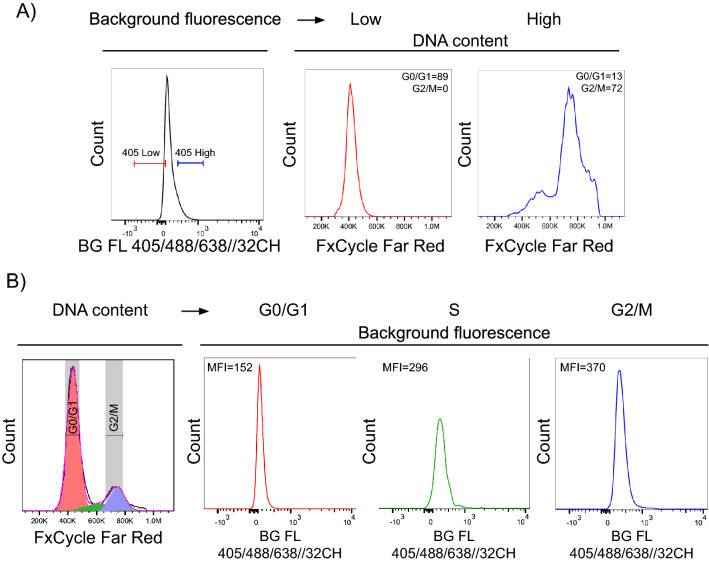
Spectral flow cytometry confirms the association of background fluorescence and cell cycle state. HCT 116 cells were stained using LIVE/DEAD Fixable Dead Cell Stain Kit, fixed in 4% PFA and DNA was labelled using FxCycle Far Red Stain. The background fluorescence was measured in the range of 420–800 nm using 32 detectors with a spectral analyzer (SONY SP6800). (**A**) Representative image of background fluorescence and cell cycle profile of HCT 116 cells detected after simultaneous 405, 488, and 638 nm excitation. Dead cells were excluded and fractions of cells with low (bottom 10%) and high (top 10%) background fluorescence were gated for DNA content (cell cycle) analysis. The values of G0/G1 and G2/M phase represent the proportion of cells. (**B**) Examples of reversed gating strategy, when modelled cell phases (FlowJo) were gated and analyzed for background fluorescence. Median fluorescence intensity (MFI) was then calculated for each phase. Data are representative from two independent repetitions. For total DNA content distribution of the whole population, see Supplementary Fig. 8B.

Differences in the cell cycle stage of sorted cells can have profound effect on downstream analyses. To systematically test whether the distribution of background signals, or autofluorescence, of cells analyzed with flow cytometry relates to their cell cycle status, we first analyzed the cell cycle profile of lower and upper 10% of cells gated based on their background fluorescence. We labelled two cell lines, HCT 116 (human colon cancer) and cE2 (murine prostate cancer) with a series of commonly used DNA stains in both native (Hoechst 33342) and fixed states (DAPI or propidium iodide). We recorded their fluorescence at a single cell level using flow cytometry across all detectors, including the empty, background channels. We then focused on these background channels and applied a back-gating strategy, separating the bottom 10% of the lower intensity population and the top 10% of the higher intensity population in background fluorescence channels (Fig. 1). To control for a possible fluorescence spillover effect, the background fluorescence was assessed on different optical line of the instrument (Sup. Table 1) than the one used for DNA dye excitation/detection. Our results showed that in both cell lines, in both native and fixed detection conditions, and in all DNA dye conditions, the population of cells with “low” background fluorescence intensity was enriched in cells that were in G0/G1 phase of their cell cycle. Inversely, cells selected based on the “high” background fluorescence intensity were dominated by the cell population in the G2/M phase (Fig. 1, Sup. Anim. 1A and 1B). This phenomenon was observed on a conventional flow cytometer using the three most commonly used lasers (wavelengths 405, 488, and 639 nm) and detecting background fluorescence on three detectors with spectral bandpasses of 450/50, 525/50, and 780/60 (Fig. 1 and Sup. Fig. 1). To extend these observations across the full detection spectrum, we performed similar analysis using spectral flow cytometry (see “Material and methods” section for details). This approach allowed us to subtract the signal from the DNA dye (FxCycle Far Red Stain) and observe the total fluorescent background of HCT 116 cells over the entire wavelength range. We defined the “lower” and “upper” fraction background cells, similarly to conventional cytometry, in all 32 channels simultaneously. We then used two approaches to analyze the data: First, we visualized the population of cells with “low” and “high” background in the cell cycle parameter as we did for conventional flow cytometry (Fig. 2A). Second, we compared the background fluorescence of cell populations at different phases of the gated cell cycle based on the amount of DNA labeled with the DNA dye (Fig. 2B). Both approaches confirmed our observations that the fraction of cells with “low” levels of background fluorescence represents cells predominantly in the G0/G1 phase of the cell cycle, while cells with “high” background fluorescence reside predominantly in the G2/M phase (G0/G1: 89% vs. 13%, G2/M: 0% vs. 72%; G0/G1 background MFI 152 vs. G2/M background MFI 370, Fig. 2A,B). Moreover, we provide evidence that this phenomenon is spectrally independent and can be observed in all fluorescent channels used in conventional and spectral flow cytometers. With these experiments we show that the background fluorescence, as assessed with flow cytometry, shows an association with cell cycle, with highly autofluorescent cells being enriched in cells in later stages of cell cycle.

### Experimental modulation of cell cycle progression affects background autofluorescence of the cells

**Figure 3:**
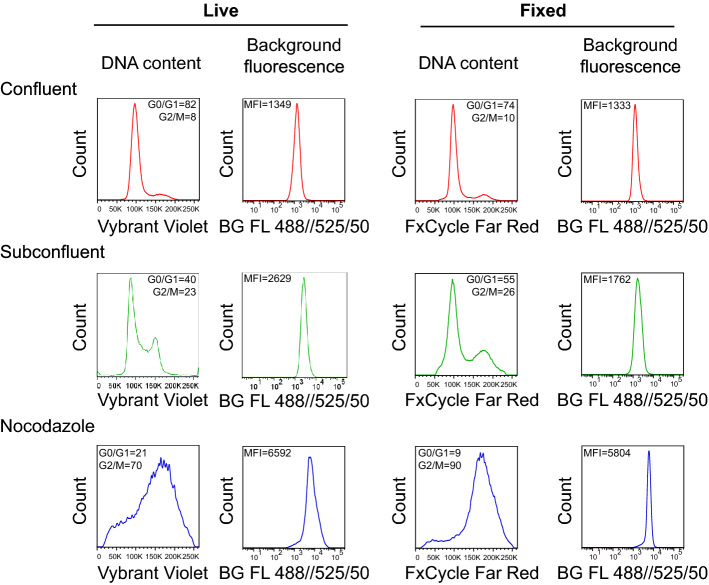
Experimental modulation of cell cycle progression affects the background autofluorescence. HCT 116 cells were synchronized to G0/G1 phase by cultivation to the full confluency (red line plots) or arrested in the G2/M phase with nocodazole treatment (blue line plots). Control HCT 116 cells were cultivated in standard subconfluent conditions (green line plots; see Methods for details). Dead cells were excluded from analysis using LIVE/DEAD Fixable Dead Cell Stain Kit. The cell cycle was then analyzed in both native (Vybrant DyeCycle Violet) and fixed (FxCycle Far Red Stain) conditions. Together with background fluorescence. The numbers of G0/G1 and G2/M phases represent a percentage of cells. The values of G0/G1 and G2/M phase represent the proportion of cells. Data are representative from two independent repetitions. *MFI* median fluorescence intensity.

Whether the background fluorescence reflects the state of cultured cells remains, in the context of cell cycling, largely unknown. To demonstrate that the cell cycle distribution does indeed affect the background fluorescence intensity, we used several experimental strategies to perturb the cell cycle progression in the HCT 116 cells in vitro. We compared cells collected in the subconfluent state of cell culture (control, asynchronously proliferating) with the cells that are in fully confluent state (predominantly in the G0/G1 phase), and cells that are arrested in the G2/M phase after nocodazole treatment (commonly used synchronization technique)^25,26^. In both native and fixed states, fully confluent cells showed lower background fluorescence compared to the subconfluent cells (native background fluorescence MFI 1349 vs. 2629). On the other hand, cells with nocodazole-induced cell cycle arrest in the G2/M phase showed a significant increase in their background fluorescence (native background fluorescence MFI 2629 vs. 6592, Fig. 3). Taken together, the cell cycling reflects on background fluorescence of analyzed cell population and vice versa.

### Characterization of low and high background autofluorescence fractions of the cells

**Figure 4:**
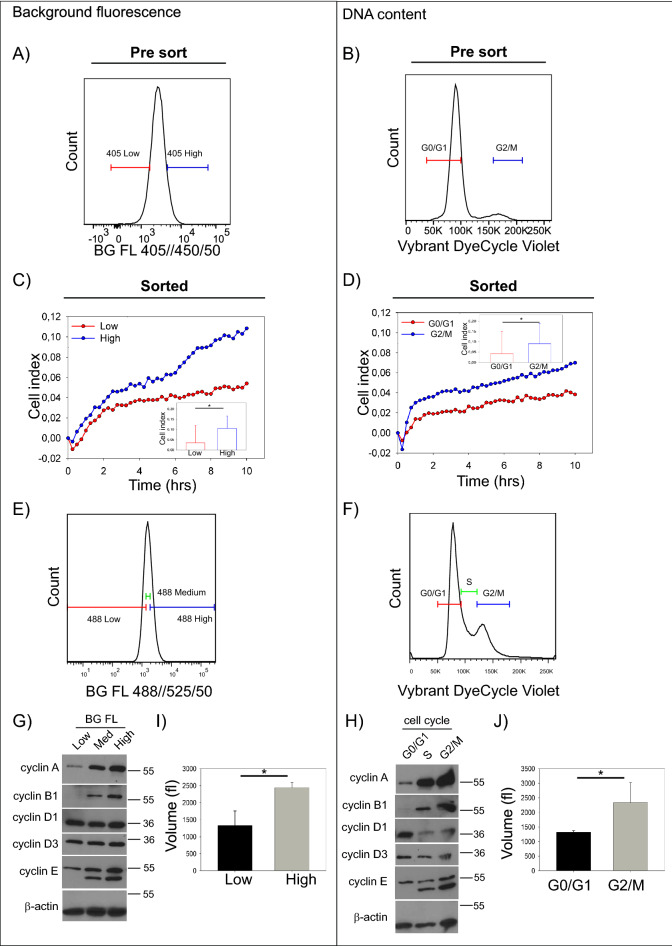
Assessment of low and high background autofluorescence cell fractions in vitro. Representative figure showing fractions selected for direct functional comparison of live HCT 116 cells sorted based on 10% low and 10% high background fluorescence (**A**) or G0/G1 and G2/M cell cycle phases after staining with cell-penetrant, native DNA dye (Vybrant DyeCycle Violet), (**B**). Sorted cell fractions were subjected to real-time cell adhesion monitoring with signal being recorded every 15 min and cell index being a function of cell adhesion. The adhesion pattern of cells sorted based on the extent of their background fluorescence (**C**) resemble cells sorted based on their corresponding cell cycle phase (**D**). Data are pooled from three technical replicates *per* condition and three independent experiments are shown, for details see Methods (*~ *P* < 0.05 for cell index at 10 h), see Sup. Fig. 2 for post-sorting purity assessment. Similarly, distribution of selected cyclins is similar between cells sorted based on their background fluorescence (**E**,**G**) or cell cycle phase (**F**,**H**). Representative blots are from two independent replicates, uncropped membrane scans are provided in Sup. Fig. 7. Lastly, cell volume as determined with CASY TT follows the same pattern for cells sorted based on their background fluorescence (**I**) or cell cycle phase (**J**). Data pooled from three independent experiments and plotted as mean ± S.D. (*~ *P* < 0.05).

We next wanted to empirically validate the association between cell cycle state and background fluorescence. To achieve this, we performed a more detailed characterization of cell populations sorted based on “low” and “high” background fluorescence (Fig. 4A). In parallel, we sorted cells based on their cell cycle state (Fig. 4B) and performed similar characterization. One of the functional differences between cells in the cell cycle interphase and mitosis is linked to the cell adhesion^27^. Therefore, we used sorted cell fractions and performed cell adhesion assay using a label-free, real-time, impedance-based system^28,29^. Our data showed significant differences in the cell adhesion between fractions sorted based on “low” and “high” background fluorescence (Fig. 4C). Similarly, we observed analogous pattern for cell fractions sorted based on their cell cycle state, i.e. G0/G1 versus G2/M (Fig. 4D). We further performed analysis of protein content in sorted fractions, focusing on the key components of cell cycle regulation—cyclins^30^. We sorted cells based on their “low”, “medium”, and “high” background fluorescence (Fig. 4E), or cell cycle phases after native, cell-penetrant DNA dye staining (Vybrant DyeCycle Violet, Fig. 4F; post-sort purity shown in Sup. Fig. 2A, B was assessed before the sample lysis). Analysis of cyclin expression confirmed the expected pattern in the samples sorted based on cell cycle phase (Fig. 4H). Strikingly, we observed an almost identical pattern of cyclin distribution in fractions sorted based on their background fluorescence intensity (Fig. 4G). We next hypothesized that the increase in cell autofluorescence in G2/M is related to a concomitant increase in cell volume/size. To test this, we analyzed the volume of cells from sorted cell fractions using an electronic cell counter and analyzer system, CASY TT. Our data showed the expected differences between “low” versus “high”, and G0/G1 versus G2/M sorted fractions. This analysis confirmed the size similarity between “low” and G0/G1 sorted fractions, and between “high” and G2/M sorted fractions (Fig. 4I,J respectively). Our characterization of the cell fractions sorted based on their background fluorescence showed intriguing similarity to the cells sorted based on their cell cycle phase. These results provide additional evidence for a direct relationship between cell cycle state and background fluorescence intensity.

### Association of cell size and background fluorescence is reproducible on different flow cytometers

**Figure 5:**
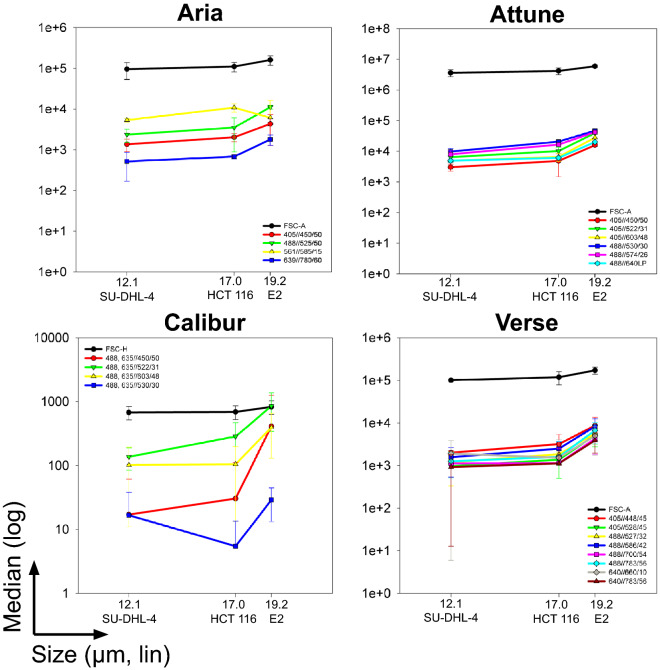
Average cell size correlates with the background fluorescence intensity. The size of the three different cell lines SU-DHL-4 (average diameter 12 µm), HCT 116 (17 µm), and E2 (19 µm) was determined in suspension using the CASY TT cell counter. The unlabeled cells were then analyzed using four different flow cytometers and median background fluorescence for all available lasers (BD FACSAria II SORP) or detectors (TFS Attune, BD FACSCalibur, BD FACSVerse) was determined. Data are plotted as median ± S.D. from at least three biological replicates.

Since the generalization of our observation was unknown, we aimed to address the reproducibility and robustness of the association between cell cycle/cell size and intensity of background autofluorescence. We performed additional measurements and analyses that involved several routinely used, state-of-the-art flow cytometers and several cell lines with different cell sizes, growth conditions, and species of origin. We included a human lymphoblast-like cell line, SU-DHL-4, that grows in suspension and hence does not require detachment from the cell culture plastic. The median diameter of SU-DHL-4, HCT 116 and E2 cell lines measured on the CASY TT system ranged from 12 to 19 µm. We analyzed the background fluorescence of these cell lines using four different flow cytometers. Our systematic assessment showed that the background fluorescence signal increases together with cell size in all channels and after different excitations, independently of the used cytometer (Fig. 5).

One of the outstanding questions that remained unanswered during these analyses was whether this phenomenon associates only with cellular objects, or whether it applies to particles in general. We analyzed polystyrene particles with specific sizes, ranging from 2 to 14.7 µm in diameter, on 5 different flow cytometers. First, we compared populations of particles with different sizes on forward and side scatter (Sup. Fig. 3A). Second, we analyzed these populations on fluorescence channels (Sup. Fig. 3B). Based on the quantified green fluorescence channel medians we confirmed that increasing particle size is associated with the increase in signal in fluorescence channels (Sup. Fig. 3C). This observation was confirmed in all fluorescence channels, and the pattern of increasing signal with particle size was also evident for all used lasers (Sup. Fig. 4A). Finally, we performed the same measurements with a spectral flow cytometer in 32 fluorescence channels, splitting the light spectra from 420 to 800 nm into small fractions (Sup. Fig. 4B). The connection between increasing fluorescence background and increasing particle size was present throughout the entire range of the 32 detectors. In summary, the relationship between cell size and background fluorescence was reproducible across different flow cytometers and can be generalized to a non-cellular particles, such as polystyrene beads.

### Distribution of the fluorescence signal within asynchronous cell population is associated with cell cycle state

**Figure 6:**
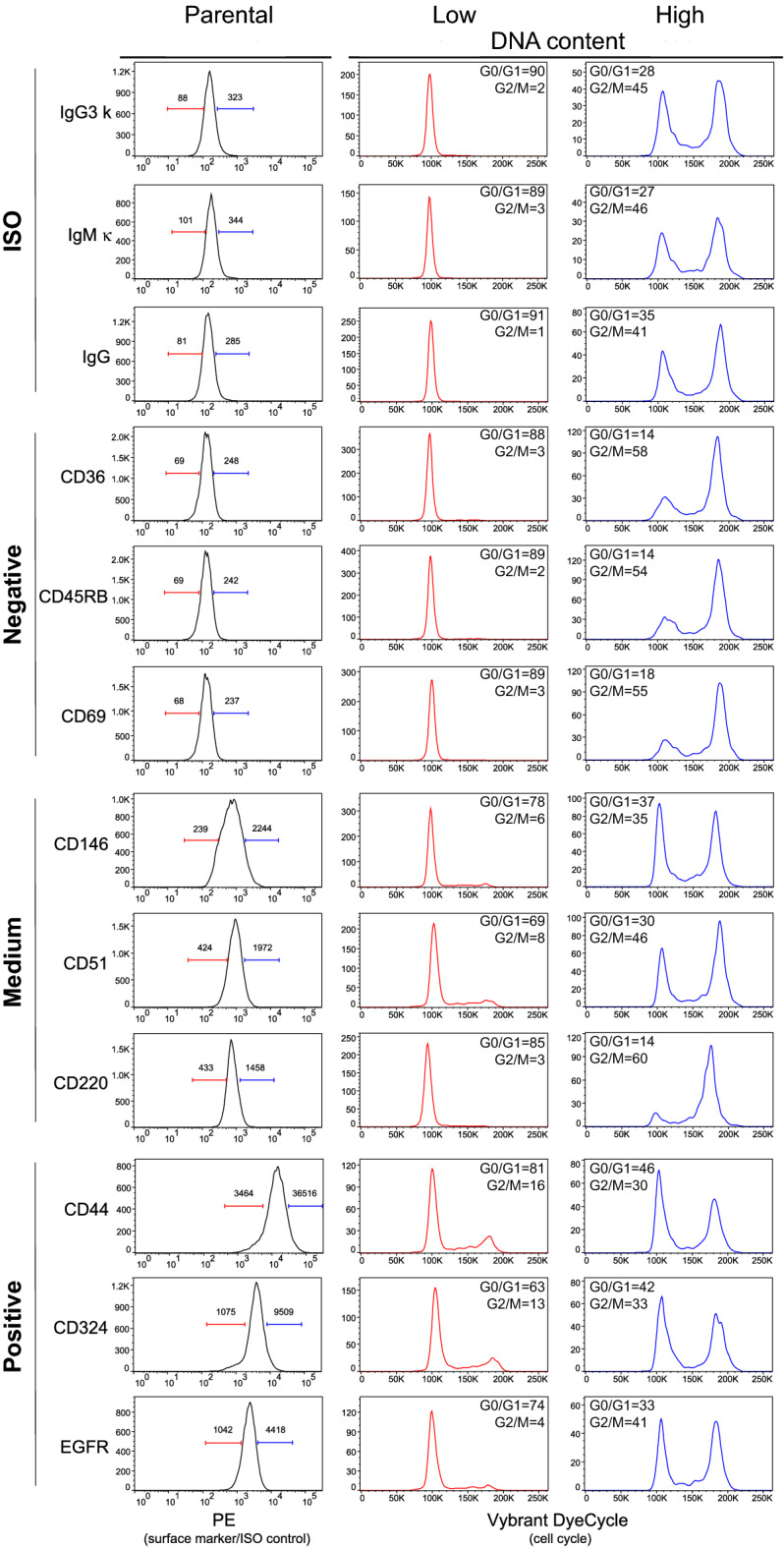
High-throughput cell surface marker screen confirms general association between fluorescence distribution and cell cycle. Histograms in the first column represent characteristic examples of isotype (negative) controls, markers with undetectable expression (< 1% positivity), medium expression (~ 50% positivity) and markers with high, positive expression (> 99% positivity). The fractions of the cells with 10% low and 10% high specific fluorescence (PE channel) intensities were gated and analyzed for cell cycle distribution and are shown in the second and third column. The values of G0/G1 and G2/M phase represent cell proportions. Values above gating lines in the first histogram column of the histograms represent median fluorescence intensities of gated cell fraction. Screen was performed with LEGENDScreen human PE kit, for details see Methods. For total DNA content distribution of the whole population and gating strategy see Sup. Fig. 5.

**Figure 7:**
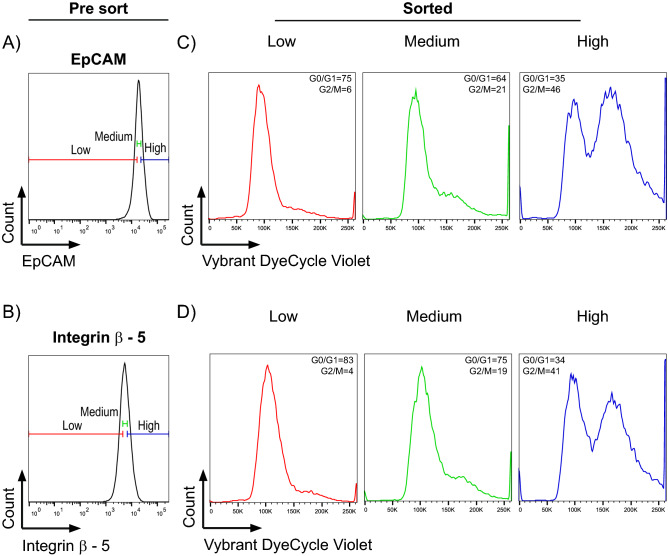
Post-sorting analysis of cell cycle distribution in the fractions of cells with different levels of specific stain fluorescence. Viable HCT 116 cells were sorted in their native state based on the low, medium, and high specific fluorescence intensity after cell surface staining for EpCAM (**A**) or integrin β5 (**B**). Sorted fractions were subsequently stained for DNA content with cell permeable DNA dye (Vybrant DyeCycle Violet) and immediately re-analyzed for cell cycle. Values for G0/G1 and G2/M phases in the sorted low, medium, and high fluorescence for EpCAM (**C**) and integrin β5 (**D**) cells represent cell proportions. The values of G0/G1 and G2/M phase represent cell proportions. Data are representative from at least three independent repetitions. For post-sort purity assessment, see Sup. Fig. 6.

Flow cytometry is used to assess the presence or quantify the amount of expression of selected antigens with fluorochrome-tagged antibodies. The next logical step was therefore to assess the effects of cell cycle on the distribution of specific immunofluorescent stain. To gain a comprehensive understanding of such relationship, we measured the expression of 332 cell surface markers and 10 isotype controls in HCT 116 cells along with the DNA staining, allowing for simultaneous cell cycle analysis. Following flow cytometric analysis, we used similar gating strategy as shown in Sup. Fig. 1 and delineated the upper and lower 10% of cells in terms of each surface marker expression. With such strategy, we examined the cell cycle distribution profile of the “low” and “high” populations in the commonly observed scenarios: (1) negative expression—antigen not present, with a signal of intensity similar to that of isotype control, (2) medium expression—weakly expressed antigen that exhibits only a “shift” in the intensity, and (3) positive expression—highly expressed antigen by the entire cell population. For each scenario, we selected a representative group of cell surface markers (Fig. 6). Direct comparison between isotype controls and the three scenarios described above confirmed that the cell cycle distribution was related to the fluorescence intensity in extensive array of antigens. The fraction of cells defined based on the lower 10% values of fluorescence intensity is mainly enriched for cells in the G0/G1 phase of the cell cycle, whereas the fraction from the upper 10% values represents mainly cells in the G2/M phase (see data on the proportion of cells in G0/G1 and G2/M in Fig. 6). This phenomenon is obvious in all categories, even in the positive population with strong specific fluorescence signals. For independent confirmation, we performed cell sorting in the native state based on the low, medium, and high fluorescence intensities of two model surface antigens, EpCAM and integrin β5 (Fig. 7A,B; see Sup. Fig. 6 for post-sort purity assessment). These sorted fractions were then stained for DNA content with Vybrant DyeCycle Violet, and reanalyzed immediately (Fig. 7C,D). The cell cycle distribution recapitulated the previously observed pattern, with low-fluorescence sorted fraction being enriched in the G0/G1 cells, medium-fluorescence population enriched in the G0/G1/S cells, and the high-fluorescence sorted population enriched in the G2/M cells (see data on the proportion of cells in G0/G1 and G2/M in Fig. 7). Overall, we provide strong evidence that the variability in the distribution of background and specific fluorescence signal is related to the cell cycle status. Orthogonally, the cell cycle distribution affects the distribution of both background and specific fluorescence signals.

## Discussion

Both cell cycle and cell volume are well-known sources of bias, introducing within-the-cell-type phenotypic and functional heterogeneity in the scRNA-seq-generated results^4–7^. With recent technological advancements in cytometry, such consideration of the cell cycle/cell volume effects in polychromatic flow cytometry data becomes necessary. Here, we describe the association between cell cycle state/cell size and the distribution of fluorescence intensity, systematically dissected by flow cytometry. First, we demonstrated that the “low” fraction of background fluorescence signal was enriched mostly in the cells in G0/G1 phase, while the “high” fraction contained cells mostly in the G2/M phase. Employing different instrumental setups, we showed that this phenomenon is spectrally independent and can be observed in all assessed fluorescent channels used in conventional and spectral flow cytometers. This relationship between cell cycle and cell size was confirmed by additional experiments in which DNA was first labeled natively, and its intensity analyzed by flow cytometry was used to determine the intensity of background fluorescence in different spectral ranks. Experimental manipulation of the cell cycle profile and subsequent analysis of autofluorescence also corroborated this relationship. For an ultimate validation, we chose to sort cells based on the intensity of background fluorescence and analyze the expression levels of key cell cycle-regulating cyclins, along with a functional approach that tested their ability to adhere to the cell culture surface. These analyses showed that the cells sorted using “low” and “high” approach differed in their ability to adhere, and these results are consistent with previously published studies in which a higher ability to adhere was demonstrated for cells in the G2/M phase of the cell cycle^27^. Moreover, the cyclin expression profile corresponded with that of cells that were sorted based on the DNA amount. The simplest explanation was that the rise in cellular autofluorescence, linked to the cell cycle progression, is related to the change in the cell size^31^. The relationship between autofluorescence and cell size has been previously demonstrated in several studies that, however, did not provide a direct link to changes in the cell cycle distribution^32–34^. We therefore confirmed that cells sorted based on high background fluorescence are typically larger in cell volume, corresponding to cells sorted based on the amount of DNA in the G2/M phase of the cell cycle. The reproducibility and robustness of these associations were addressed by measurements involving several flow cytometers and cell lines with different cell sizes. We confirmed that the relationship between cell size and background fluorescence is reproducible across different flow cytometers and is not only related to differences in cell size but is also observed for other particles, such as polystyrene beads. Finally, we confirmed that the relationship between cell cycle and background fluorescence distribution also remains valid in the case of specific fluorescence. Analysis of 342 surface molecules together with cell cycle confirmed that the variability of specific fluorescence distribution (as a measure of individual surface antigen expression) corresponded to the cell cycle distribution observed for background fluorescence. Overall, we showed that cell cycle status is related to both background and specific fluorescence signals of different abundance. Additionally, we conclude that cell cycle distribution affects the distribution of both background and specific fluorescence signals. We are aware of some of the limitations of our study, in particular, we are unable to simply distinguish between the consequences of intrinsic cell size changes and separate them from those associated with cell cycle phase changes, further recognizing that cell differentiation/maturation may be fundamentally involved in the spectrum of these changes. Nevertheless, based on the evidence presented we argue that the interpretation of data obtained solely from comparisons of populations defined in terms of symmetric fluorescence signal distribution could be misleading. Without sufficient validation, these results can be confounded by cell cycle/size distribution, and we advise further confirmation using other, complementary techniques. These observations, specifically the effect of cell cycle state and cell size, should be considered also during visualization of polychromatic flow cytometry data using t-SNE and other popular algorithms.

## Material and methods

### Cells and cell culture

The mouse prostate cancer cell line cE2 and E2^35^ (a kind gift from Dr. Pradip Roy-Burman, University of Southern California, CA, USA) was maintained in Dulbecco's modified Eagle's medium (DMEM) high glucose with GlutaMAX (32430, Gibco, Thermo Fisher Scientific, USA, TFS) supplemented with rhEGF (6 ng/mL, Sigma-Aldrich, Merck, USA), insulin (5 μg/mL, Sigma-Aldrich, Merck), bovine pituitary extract (25 μg/mL, Hammond Cell Tech, USA), penicillin (100 U/mL) and streptomycin (0.1 mg/mL; PAA, Austria), and 10% fetal bovine serum (PAA). Human colon adenocarcinoma cells HCT 116 (a kind gift from Dr. Bert Vogelstein, Johns Hopkins University, MD, USA) were maintained in McCoy's 5A (modified) medium, GlutaMAX (36600, TFS) supplemented with penicillin (100 U/mL) and streptomycin (0.1 mg/mL, TFS) and 10% heat-inactivated fetal bovine serum (TFS). Human B lymphoblasts SU-DHL-4 (a kind gift from Dr. Martin Trbušek, Masaryk University, Czech Republic) were cultured in Roswell Park Memorial Institute's medium (RPMI) 1640 with GlutaMAX (72400, TFS) and 10% fetal bovine serum (TFS), penicillin (100 U/mL) and streptomycin (0.1 mg/mL; TFS) addition^36^. All cell lines were maintained in cell culture plastic from TPP (Switzerland) or BD Falcon (BD Biosciences, CA, USA) in a humidified incubator at 37 °C in an atmosphere of 5% CO_2_. The cells were harvested by incubation in 0.05% EDTA in PBS followed by trypsinization (0.25% w/v trypsin/0.53 mM EDTA in PBS) and counted with CASY TT automatic cell counter (Innovatis AG, Germany). Cell suspensions were filtered through sterile 70- or 100-μm syringe filters (Filcons, Germany) before analysis or sorting.

### Instrumentation

Cell sortings and some of the experiments were performed on FACSAria II SORP system (BD Biosciences) equipped with five lasers (excitation wavelengths: 355, 405, 488, 561 and 639 nm, respectively). For all sortings, a 100-μm nozzle (20 psi) was used, and post sorting purity was analyzed immediately after sorting. We used four additional flow cytometers to conduct the experiments: FACSVerse (BD Biosciences), Attune (1st generation, TFS), FACSCalibur (BD Biosciences), and SP6800 spectral analyzer (SONY). The advantage of including spectral analyzer on the top of conventional flow cytometers is that it allows for more detailed spectral detection. SP6800 contains similar laser excitation sources, and we used 32 channels with narrow bandpasses starting at 420 and ending at 800 nm for detection (see Sup. Table 1 for details). A specific feature of this system is its ability to calculate a so-called virtual parameter that collects the signal from all 32 channels. Furthermore, the analyzer allows to apply a spectral unmixing algorithm, detecting signal of the other fluorescent markers in the panel. Spectral unmixing is calculated with the signal previously collected from individually stained controls over the entire 420–800 nm spectrum range. Details about the instruments’ configuration are shown in Supplementary Table 1.

### Flow cytometry staining and cell sorting

Samples of fixed HCT 116 cells stained for viability (LIVE/DEAD Fixable Violet Dead Cell Stain Kit, TFS) and cell cycle (FxCycle Far Red Stain, TFS) were analyzed on SP6800 spectral analyzer after 405 and 488 together with 638 nm excitation on 32 channels in narrow bands for fluorescence detection. Detectors covered the range from 420 to 800 nm, using SONY’s software we combined all 32 channels into one parameter “AF”. Dead cells were previously excluded. For purpose of real-time adherence monitoring, we sorted HCT 116 cells (80 K per group) in 2 repetitions based on autofluorescence on 405 nm laser and cell cycle phase (staining with Vybrant DyeCycle Violet Stain, V35003, TFS) (Fig. 3A). Dead cells were excluded by LIVE/DEAD Fixable Far Red Dead Cell Stain Kit. The purity of sorted samples was controlled prior to seeding. HCT 116 cells (800 K cells per group and repetition) were also sorted for protein analysis with western blot (see below) based on autofluorescence on 488 nm laser (Fig. 4A). Dead cells were excluded using propidium iodide. Alternatively, HCT 116 cells originated from the same flask (750 K cells per group and repetition) were sorted based on cell cycle distribution (staining with Vybrant DyeCycle Violet Stain) (Fig. 4C). The purity of sorted cells was reanalyzed on the sorter immediately after sorting (Sup. Fig. 2). HCT 116 cells were stained on viability (LIVE/DEAD Fixable Far Red Dead Cell Stain Kit) together with biotin-conjugated CD326 (EpCAM) Monoclonal Antibody (1B7) (1:200, eBioscience, TFS, cat. no. 13-9326-82) or unconjugated Purified anti-human integrin β5 Antibody (1:100, BioLegend, cat. no. 345202). For unspecific binding and secondary staining were used streptavidin FITC (1:2000, eBioscience, cat. no. 11-4317-87) or donkey anti-Mouse IgG (H + L) highly cross-adsorbed secondary antibody Alexa Fluor 488 (1:500, eBioscience, TFS, cat. no. A21202) antibodies. Cells were sorted into low, medium and high populations divided into thirds on both markers. Immediately after sorting, post sort purity was analyzed and sorted cells from each fraction were stained for DNA content using Vybrant DyeCycle Violet Stain (1:1000, Invitrogen) as described below.

### Data analysis

Cell doublets, aggregates and debris were excluded from the analysis based on a dual-parameter dot plot in which the pulse ratio (signal area/signal high; y-axis) versus signal area (x-axis) was displayed. Dead cells were excluded from the analysis by staining with propidium iodide (Sigma-Aldrich, Merck) or LIVE/DEAD Fixable Dead Cell Stain (different fluorescence reactive dyes; Invitrogen, TFS). Cytometric data were recorded using FACSDiva software (Version 6.1.3; BD Biosciences), Attune Cytometric Software (Version 2.1; TFS) and FACSuite (Version 1.0.5.3841 and 1.0.6; BD Biosciences). Data analysis was performed using FlowJo software (Version 7.6.5 and 10.0.7, BD Biosciences). List mode data are uploaded into the Flow Repository database of flow cytometry experiments (https://flowrepository.org/id/FR-FCM-ZYFP).

### Cell cycle analysis

Trypsinized and PBS-washed cE2 or HCT 116 cells were stained for cell cycle immediately in their native state, or after fixation (70% ethanol or 4% paraformaldehyde) and permeabilization (0.1% Triton‑X100). Live cells were stained in complete media with Hoechst 33342 (Sigma-Aldrich, Merck) or Vybrant DyeCycle Violet Stain (Invitrogen) for 45 min at 37 °C. Fixed and permeabilized cells were stained with Vindelov's solution^37^, DAPI, or FxCycle Far Red Stain (Invitrogen, TFS). Staining was performed for 30 min at 37 °C for Vindelov, at room temperature for DAPI, and at 4 °C for FxCycle Far Red Stain.

### Cell cycle synchronization

HCT 116 cells were synchronized in G1 and G2/M phases prior to the cell cycle staining. Cells were maintained for 8 days in the same culture dish, with media change every 2–3 days, to reach 100% confluence and synchronize in G0/G1. Subconfluent (70–80%) cells were used as a control sample. For G2/M arrest, cells were treated for 24 h with nocodazole (final concentration 100 ng/mL, Sigma-Aldrich, Merck), and only floating cells were collected for further processing.

### Cell surface markers screening

HCT 116 cells were expanded, harvested and 3 × 10^8^ cells was stained for cell cycle (Vybrant DyeCycle Violet Stain) and viability (LIVE/DEAD Fixable Far Red Dead Cell Stain Kit) and dispensed into LEGENDScreen Human Cell Screening (PE) Kit plates for surface staining with 332 cell surface markers and 10 isotype controls (cat. no. 700001, BioLegend, CA, USA). Further processing of cells was done according to the manufacturer's recommendation. Cell from each plate well were recorded on BD FACSVerse for 2 min *per* well on medium speed. Only viable (LIVE/DEAD negative), single cells (FSC-A vs. FSC-H followed by single-cell selection on Vybrant-A vs. Vybrant-W plot) without debris (FSC-A vs. SSC-A) were selected for further analysis.

### Real-time cell adherence analysis

Cell adherence of sorted cell populations was monitored in real-time using the xCELLigence real-time cell analysis (RTCA) DP system in combination with E-plate View inserts, equipped with the RTCA Software v1.2 (Acea Biosciences, USA). Adherence was inferred by the measurement of electrical impedance across microelectrodes that integrated into the apical surface of the well bottom of E-plates^38^. Every cell attached to microelectrodes acts as electrical insulator in conductive cell culture media and is measured as an increase in total impedance. First, a standard background measurement was recorded using 200 µL of complete culture medium every minute for 5 min. Next, 20,000 of sorted HCT 116 cells were seeded manually per each well (in multiplicate of 3 wells for each subpopulation of G0/G1, G2/M phase, 10% low and 10% highly background fluorescent cells). We then used cell index, which represents normalized electrical impedance, to reflect the cell adherence. Impedance signal was recorded continually every 15 min for up to 10 h.

### Cell volume measurement

Cells from different populations (G0/G1, G2/M phase cells, 10% “low” and 10% “high” background fluorescent cells—channel 405//450/50) were sorted as described above and analyzed on CASY TT cell counter for cell volume and viability. At least 800 cells *per* replicate were analyzed. For sorted HCT 116 fractions in Fig. 4 at least 250 cells were analyzed.

### SDS-PAGE and western blot analysis

Sorted cells were briefly spun, and cell pellets were flash frozen on dry ice, stored at − 80 °C, and then thawed on ice and lysed in radioimmunoprecipitation assay buffer (RIPA) with the addition of Protease inhibitor mix G (3910102, Serva) and Phosphatase inhibitor mix II (39055.02, Serva, Germany). RIPA was prepared fresh in-house and consisted of 150 mM NaCl; 50 mM Tris/HCl, pH 7.4; 1% Igepal CA-630 (I8896, Sigma-Aldrich, Merck); and 0.25% sodium deoxycholate (D6750, Sigma-Aldrich, Merck). Lysates were briefly sonicated, cleared, and the concentration of proteins was assessed using DC Protein Assay Kit (BioRad, CA, USA). Lysate concentrations were adjusted so they were all equal by dilution with RIPA and mixed with 5 × Laemmli loading dye (final: 2% SDS; 50 mM Tris, pH 6.8; 0.02 bromophenol blue; 100 mM DTT; 1% glycerol). Samples were boiled for 10 min at 90 °C and 10 µg of proteins were loaded. Proteins were separated by SDS-PAGE using Hoefer miniVE vertical electrophoresis unit), blotted onto PVDF Immobilon P Transfer Membrane (IPVH00010, Millipore, Merck) and blocked in 5% nonfat dry milk, pH 7.2 in TBS (20 mM Tris–HCl pH 7.2; 140 mM NaCl containing 0.05% Tween-20) for 1 h at room temperature. Membranes were incubated with following primary antibodies at 4 °C overnight: cyclin A (1:500 in 5% milk, sc-751, Santa Cruz Biotechnology, CA, USA, SCBT); cyclin B1 (1:300 in 5% BSA, sc-245, SCBT); cyclin D1 (1:500 in 5% milk, sc-20044, SCBT); cyclin D3 (1:500 in 5% milk, sc-182, SCBT); cyclin E (1:500 in 5% milk, sc-481, SCBT). Following secondary antibodies were used: ECL anti-mouse HRP linked whole antibody (1:3000 in 5% milk, NA931, GE Healthcare Biosciences) and ECL anti-rabbit HRP linked whole antibody (1:3000 in 5% milk, NA934, GE Healthcare Biosciences). Chemiluminescent signals were detected using Immobilon Western HRP Substrate (WBKLS05000, Millipore, Merck) and visualized on X-ray films (Agfa, Germany). Detection of ß-actin (1:4000 in 5% milk, A5441, Sigma-Aldrich, Merck) served as a control of equal loading. Blotting membranes were cut prior to hybridization with the antibodies, scans of stained membranes with visible protein ladders and edges, in their entirety, are presented in Supplementary Fig. 7.

### Particle size analysis

The mixture of PBS and polystyrene particles of all sizes from (Sphero Particle Size Standard Kit, Spherotech) was prepared by dispensing 2 drops of each particle size into 1 mL PBS. Background fluorescence of suspended particles was recorded on following flow cytometers: BD FACSAria II SORP, TFS Attune (1st gen.), BD FACSCalibur, BD FACSVerse, and SONY SP6800 spectral analyzer in different fluorescent channels at low speed (at least 50,000 events were recorded). Pellets from E2, HCT 116, and SU-DHL-4 cell lines were prepared as described above. The mean cell diameter for each cell line was quantified with CASY TT cell counter. Measurement of background fluorescence for each cell line was then performed on all 4 cytometers. Standardized suspension of each cell line was used for this analysis (2 million cells per 1 mL of PBS).

### Data reproducibility and statistical analysis

For the high-throughput antibody-based screen, HCT 116 cell line was analyzed one well per antibody. The initial screen was performed once. All further cell line-based experiments were performed independently at least three times. The percentage of G0/G1 and G2/M phases were calculated using Dean-Jett-Fox modelling in FlowJo v10.7.2 (BD Biosciences). Statistical analyses were performed in GraphPad Prism v9.2 (GraphPad Software, USA). Plotting and analysis was performed in SigmaPlot for Windows (Version 10.0, Systat Software). *P* values were calculated with paired t-test and ratio paired t-test (two-tailed), if not stated otherwise.

## Supplementary Information

Supplementary Legends.

Supplementary Figures.

Supplementary Table S1.

Supplementary Information 1.

Supplementary Information 2.

## Data Availability

All data generated or analyzed during this study are included in this published article (and its Supplementary Information file). List mode data were deposited to the Flow Repository database of flow cytometry experiments (https://flowrepository.org/id/FR-FCM-ZYFP). Additional raw data files are available from the corresponding authors upon reasonable request.

## References

[1] Pauklin S, Vallier L (2013). The cell-cycle state of stem cells determines cell fate propensity. Cell.

[2] Xia X, Owen MS, Lee REC, Gaudet S (2014). Cell-to-cell variability in cell death: Can systems biology help us make sense of it all?. Cell Death Dis..

[3] Pernicova Z (2014). The role of high cell density in the promotion of neuroendocrine transdifferentiation of prostate cancer cells. Mol. Cancer.

[4] Buettner F (2015). Computational analysis of cell-to-cell heterogeneity in single-cell RNA-sequencing data reveals hidden subpopulations of cells. Nat. Biotechnol..

[5] Padovan-Merhar O (2015). Single mammalian cells compensate for differences in cellular volume and DNA copy number through independent global transcriptional mechanisms. Mol. Cell.

[6] Rapsomaniki MA (2018). Cell CycleTRACER accounts for cell cycle and volume in mass cytometry data. Nat. Commun..

[7] Barron M, Li J (2016). Identifying and removing the cell-cycle effect from single-cell RNA-Sequencing data. Sci. Rep..

[8] Liu J, Fan Z, Zhao W, Zhou X (2021). Machine intelligence in single-cell data analysis: Advances and new challenges. Front. Genet..

[9] Sahir F, Mateo JM, Steinhoff M, Siveen KS (2020). Development of a 43 color panel for the characterization of conventional and unconventional T-cell subsets, B cells, NK cells, monocytes, dendritic cells, and innate lymphoid cells using spectral flow cytometry. Cytom. Part.

[10] Park LM, Lannigan J, Jaimes MC (2020). OMIP-069: Forty-color full spectrum flow cytometry panel for deep immunophenotyping of major cell subsets in human peripheral blood. Cytom. Part A.

[11] Brummelman J (2019). Development, application and computational analysis of high-dimensional fluorescent antibody panels for single-cell flow cytometry. Nat. Protoc..

[12] Mazza EMC (2018). Background fluorescence and spreading error are major contributors of variability in high-dimensional flow cytometry data visualization by t-distributed stochastic neighboring embedding. Cytom. Part A.

[13] Miranda-Lorenzo I (2014). Intracellular autofluorescence: A biomarker for epithelial cancer stem cells. Nat. Methods.

[14] Larcher V (2018). An autofluorescence-based method for the isolation of highly purified ventricular cardiomyocytes. Cardiovasc. Res..

[15] Shah AT, Cannon TM, Higginbotham JN, Coffey RJ, Skala MC (2017). Autofluorescence flow sorting of breast cancer cell metabolism. J. Biophoton..

[16] Chacko JV, Eliceiri KW (2019). Autofluorescence lifetime imaging of cellular metabolism: Sensitivity toward cell density, pH, intracellular, and intercellular heterogeneity. Cytom. A.

[17] Bagri-Manjrekar K (2018). *In*
*vivo* autofluorescence of oral squamous cell carcinoma correlated to cell proliferation rate. J. Cancer Res. Ther..

[18] Croce AC, Bottiroli G (2014). Autofluorescence spectroscopy and imaging: A tool for biomedical research and diagnosis. Eur. J. Histochem. EJH.

[19] Mosiman VL, Patterson BK, Canterero L, Goolsby CL (1997). Reducing cellular autofluorescence in flow cytometry: An in situ method. Cytometry.

[20] Kolenc OI, Quinn KP (2018). Evaluating cell metabolism through autofluorescence imaging of NAD(P)H and FAD. Antioxid. Redox Signal..

[21] You S (2018). Intravital imaging by simultaneous label-free autofluorescence-multiharmonic microscopy. Nat. Commun..

[22] Tu H (2016). Stain-free histopathology by programmable supercontinuum pulses. Nat. Photon..

[23] Kanchwala N, Kumar N, Gupta S, Lokhandwala H (2018). Fluorescence spectroscopic study on malignant and premalignant oral mucosa of patients undergoing treatment: An observational prospective study. Int. J. Surg..

[24] Wizenty J (2018). Autofluorescence: A potential pitfall in immunofluorescence-based inflammation grading. J. Immunol. Methods.

[25] Harper JV, Humphrey T, Brooks G (2005). Cell Cycle Control: Mechanisms and Protocols.

[26] Langan TJ, Rodgers KR, Chou RC, Banfalvi G (2017). Cell Cycle Synchronization: Methods and Protocols.

[27] Jones MC, Zha J, Humphries MJ (2019). Connections between the cell cycle, cell adhesion and the cytoskeleton. Philos. Trans. R. Soc. B Biol. Sci..

[28] Vistejnova L (2009). The comparison of impedance-based method of cell proliferation monitoring with commonly used metabolic-based techniques. Neuroendocrinol. Lett..

[29] Slabakova E (2015). Opposite regulation of MDM2 and MDMX expression in acquisition of mesenchymal phenotype in benign and cancer cells. Oncotarget.

[30] Lim S, Kaldis P (2013). Cdks, cyclins and CKIs: Roles beyond cell cycle regulation. Development.

[31] Amodeo AA, Skotheim JM (2016). Cell-size control. Cold Spring Harbor Perspect. Biol..

[32] Tzur A, Moore JK, Jorgensen P, Shapiro HM, Kirschner MW (2011). Optimizing optical flow cytometry for cell volume-based sorting and analysis. PLoS ONE.

[33] Bertolo A, Baur M, Guerrero J, Pötzel T, Stoyanov J (2019). Autofluorescence is a reliable in vitro marker of cellular senescence in human mesenchymal stromal cells. Sci. Rep..

[34] Schaue D, Ratikan JA, Iwamoto KS (2012). Cellular autofluorescence following ionizing radiation. PLoS ONE.

[35] Liao C-P (2007). Mouse models of prostate adenocarcinoma with the capacity to monitor spontaneous carcinogenesis by bioluminescence or fluorescence. Cancer Res..

[36] Tao K, Fang M, Alroy J, Sahagian GG (2008). Imagable 4T1 model for the study of late stage breast cancer. BMC Cancer.

[37] Vindelov LL (1977). Flow microfluorometric analysis of nuclear DNA in cells from solid tumors and cell suspensions. A new method for rapid isolation and straining of nuclei. Virchows Arch. B Cell Pathol..

[38] Staršíchová A (2009). Dynamic monitoring of cellular remodeling induced by the transforming growth factor-β1. Biol. Proced. Online.

