# Supplementary material for: Variability of fluorescence intensity distribution measured by flow cytometry is influenced by cell size and cell cycle progression
Source: Sci Rep. 2023 Mar 25;13:4889. doi: 10.1038/s41598-023-31990-1 (PMC10039904; doi:10.1038/s41598-023-31990-1)
Supplement: Supplementary file 1 — Supplementary Legends. [file 41598_2023_31990_MOESM1_ESM.docx]

**Supplementary Legends**

**Supplementary figure 1**

Background fluorescence of mouse cE2 cell line detected after 488 and 639 nm laser excitation. cE2 cells were stained using DNA dyes at fixed (DAPI) or native (Hoechst 33342) conditions. Background fluorescence was analyzed using 488 and 639 nm lasers on the optical line and detectors (565 up to 735 nm) separated from the optical line for a particular DNA dye. Samples were analyzed using flow cytometry (BD FACSAria II SORP). Dead cells were gated out, fractions of the cells with low (10%) and high (10%) background fluorescence were gated and an analysis of DNA content (cell cycle) was performed. The numbers of G0/G1 and G2/M phases represent a percentage of cells. Data demonstrate representative flow cytometry plots from at least three independent repetitions.

**Supplementary figure 2**

Analysis of purity of the samples sorted on background fluorescence, A) and cell cycle, B). Cells were processed as described in Fig. 4. Post-sorting purity was measured and analyzed at the identical setup, at least 5.000 cells for each sample were acquired. Data demonstrate representative flow cytometry plots from at least three biological replicates.

**Supplementary figure 3**

The size of the polystyrene particles correlates with the background fluorescence intensity. Mixtures of polystyrene particles (Sphero™ Particle Size Standard Kit) with a specific diameter (2 - 14.7 µm) were analyzed using four different flow cytometers. A specific population of the beads were gated and identified based on the FSC and SSC, A). The gated population were plotted and overlaid on the green fluorescence channel (Attune 530/30, Aria 525/50, Calibur 450/50, Verse 527/32), B) and the median of background fluorescence is presented in the table, C). Data in the plots demonstrate examples from at least three independent measurements.

**Supplementary figure 4**

The size of the polystyrene particles correlates with the background fluorescence intensity in various ranges of wavelengths. Polystyrene particles mixture with defined size from 2 µm up to 14,7 µm (Sphero™ Particle Size Standard Kit) identified based on the FSC vs. SSC signal (shown in Supplementary Figure 3A) were analyzed using four different flow cytometers and median background fluorescence for all available lasers (BD FACSAria II SORP) or detectors (TFS Attune, 1^st^ gen., BD FACSCalibur, BD FACSVerse) was determined, A). Data in the plots demonstrate median fluorescence intensity and are examples from at least three independent measurements. The same particles were analyzed using a spectral analyzer (SONY SP6800), B). Data in the plot represent median fluorescence intensity for each population of the beads measured in the range of 420-800 nm using 32 detectors. Example from at least three independent measurements.

**Supplementary figure 5**

Gating strategy and DNA content for LEGENDScreen data analysis. Plots represent the gating strategy before background fluorescence gating and cell cycle analysis displayed in Figure 6. First, only viable cells were selected based on the signal from the LIVE/DEAD Fixable marker, next single cells were gated on FSC-A vs. FSC-H plot. Debris was excluded from analysis using FSC-A vs. SSC-A visualization. The purity for single-cell analysis was improved using Vybrant DyeCycle Violet-A vs. Vybrant DyeCycle Violet-W distribution. Plot of total DNA content of previous population with percentage of G0/G1 and G2/M cell cycle phase is shown.

**Supplementary figure 6**

Analysis of purity of the samples sorted on specific fluorescence of EpCAM, A) and ITGB5, B). Post-sorting purity was measured and analyzed at the identical setup, at least 1.300 cells for each sample were acquired. Data demonstrate representative flow cytometry plots from at least three biological replicates.

**Supplementary figure 7**

Membrane scans related to Fig. 4G, H.

**Supplementary figure 8**

Total DNA content of live, single cells without debris related to Figure 1 is presented on panel A and for cells from Figure 2A is shown on panel B with percentage of G0/G1 and G2/M cell cycle phase.

**Supplementary Animation 1**

Visualization of correlation between cell size, cell cycle and background fluorescence. Relative cell size on forward scatter is linked to the DNA content distribution (panel A). Distribution on FSC from left to right: smaller cells dominate the G0/G1 phase, while bigger cells are enriched in the S/G2/M cell cycle phase. Similarly, DNA content distribution using DAPI staining is connected to the background fluorescence on 488//586/42 detector (panel B). DNA content distribution from left to right: Cells in the G0/G1 cell cycle phase have lower intensity background fluorescence, while cells in the S/G2/M phase have higher intensity background fluorescence.
