# Supplementary material for: Variability of fluorescence intensity distribution measured by flow cytometry is influenced by cell size and cell cycle progression
Source: Sci Rep. 2023 Mar 25;13:4889. doi: 10.1038/s41598-023-31990-1 (PMC10039904; doi:10.1038/s41598-023-31990-1)
Supplement: Supplementary file 2 — Supplementary Figures. [file 41598_2023_31990_MOESM2_ESM.pdf]

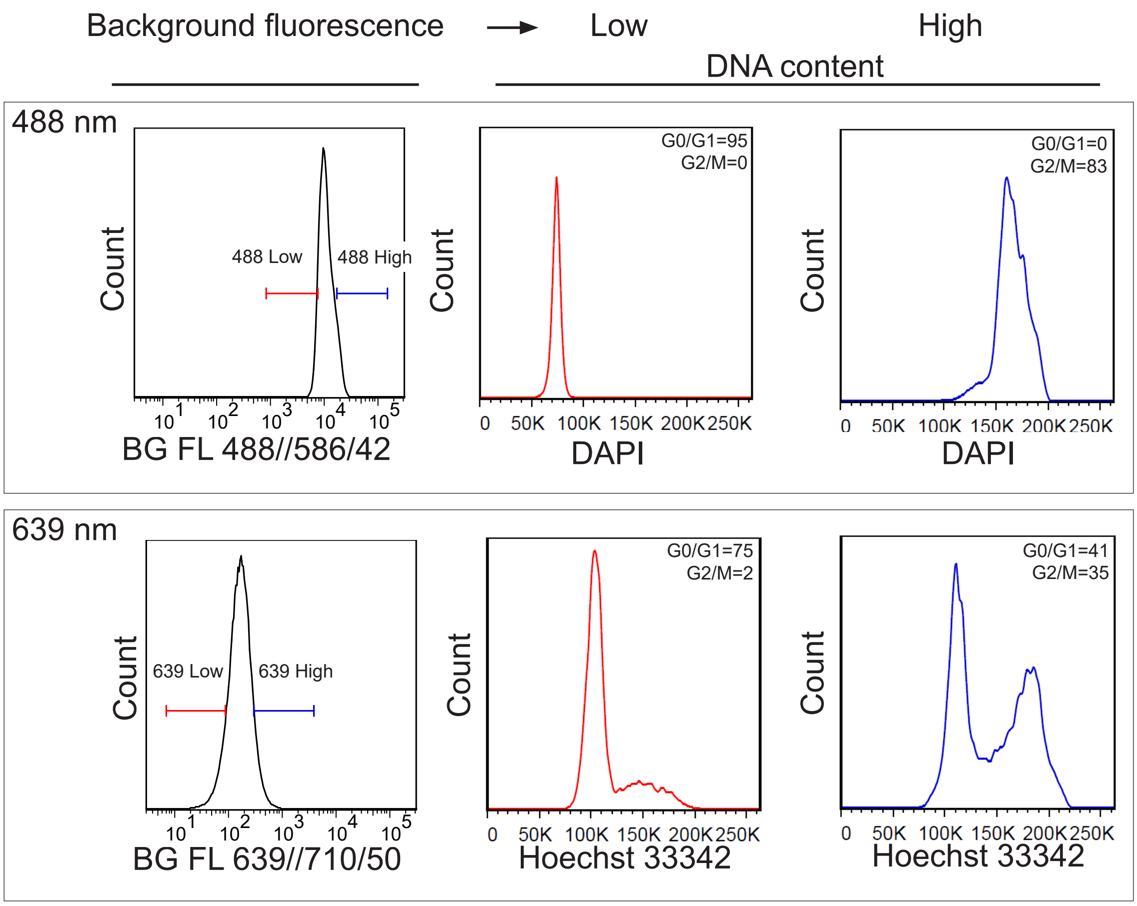

Supplementary Figure 1

Post sorting

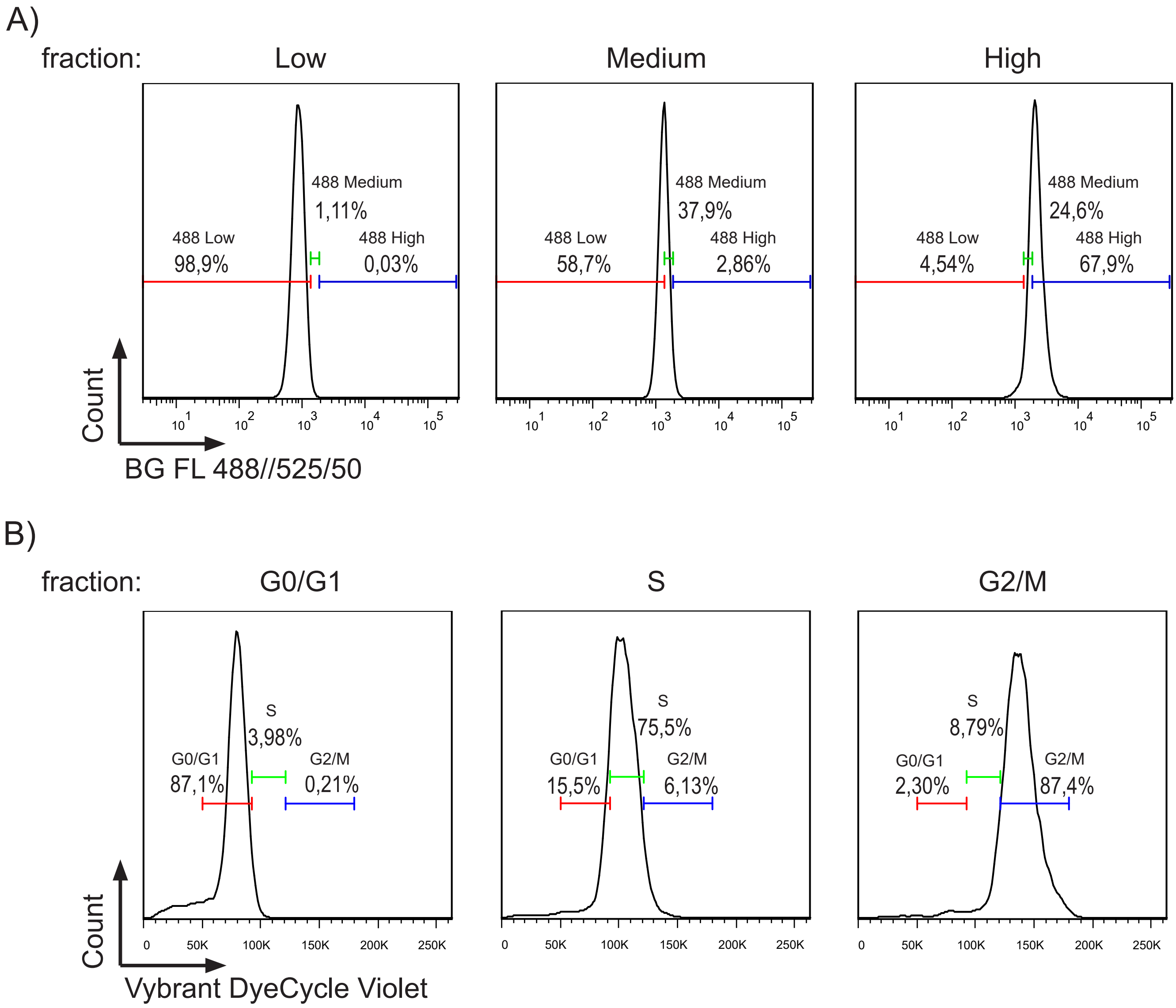

Supplementary Figure 2

A)

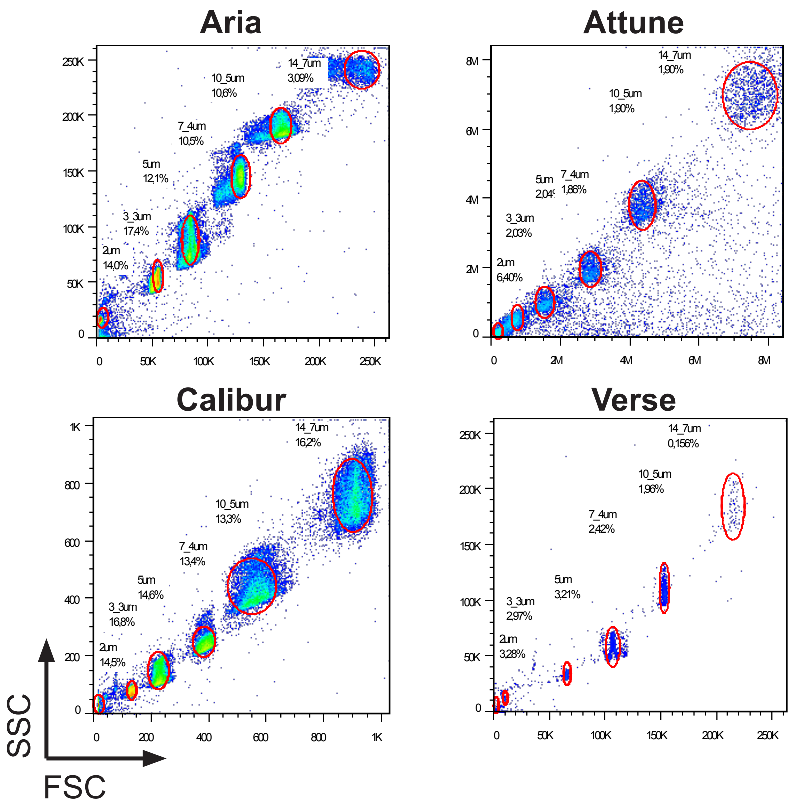

B)

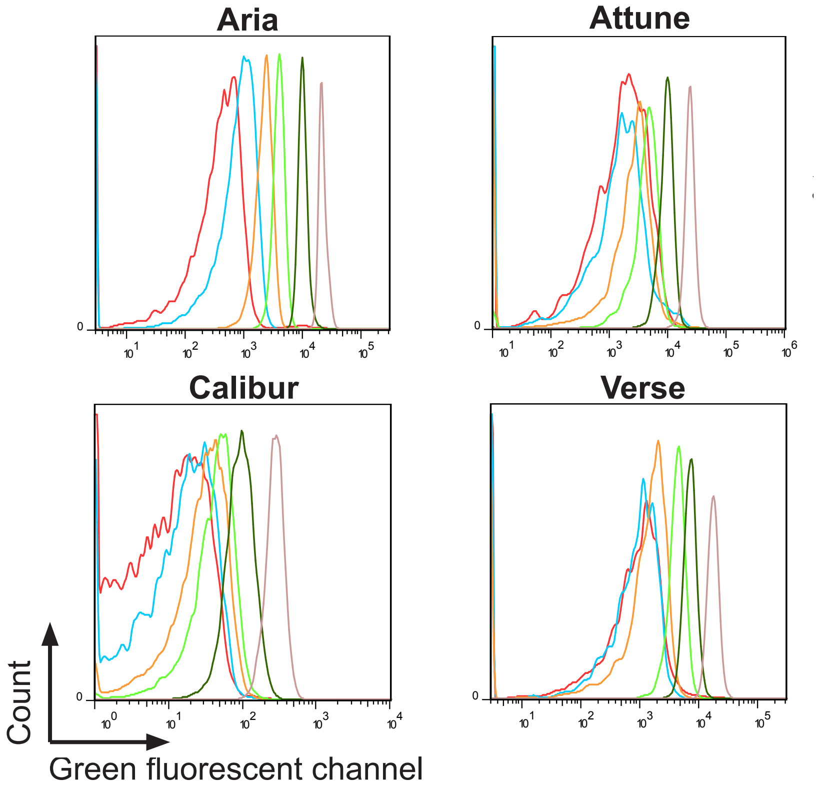

C)

Population name	Median of green fluorescent channel			
	Aria	Attune	Calibur	Verse
14.7 µm	21300	23900	275.0	17500
10.5 µm	9902	9688	92.9	7075
7.4 µm	3943	4471	44.3	4340
5.0 µm	2248	2558	30.8	1467
3.3 µm	804	981	16.6	763
2.0 µm	296	369	8.51	614

Supplementary Figure 3

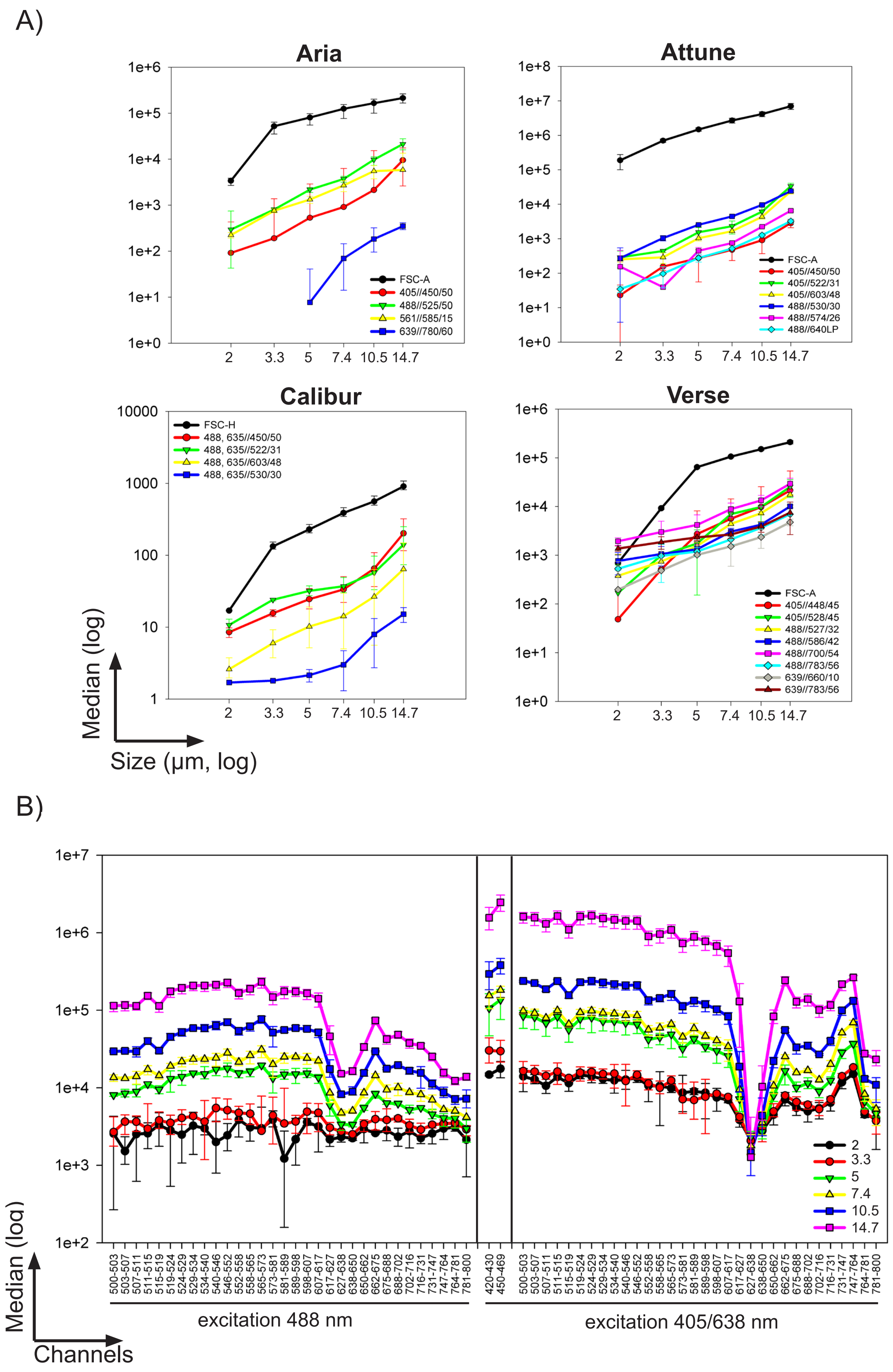

Supplementary Figure 4

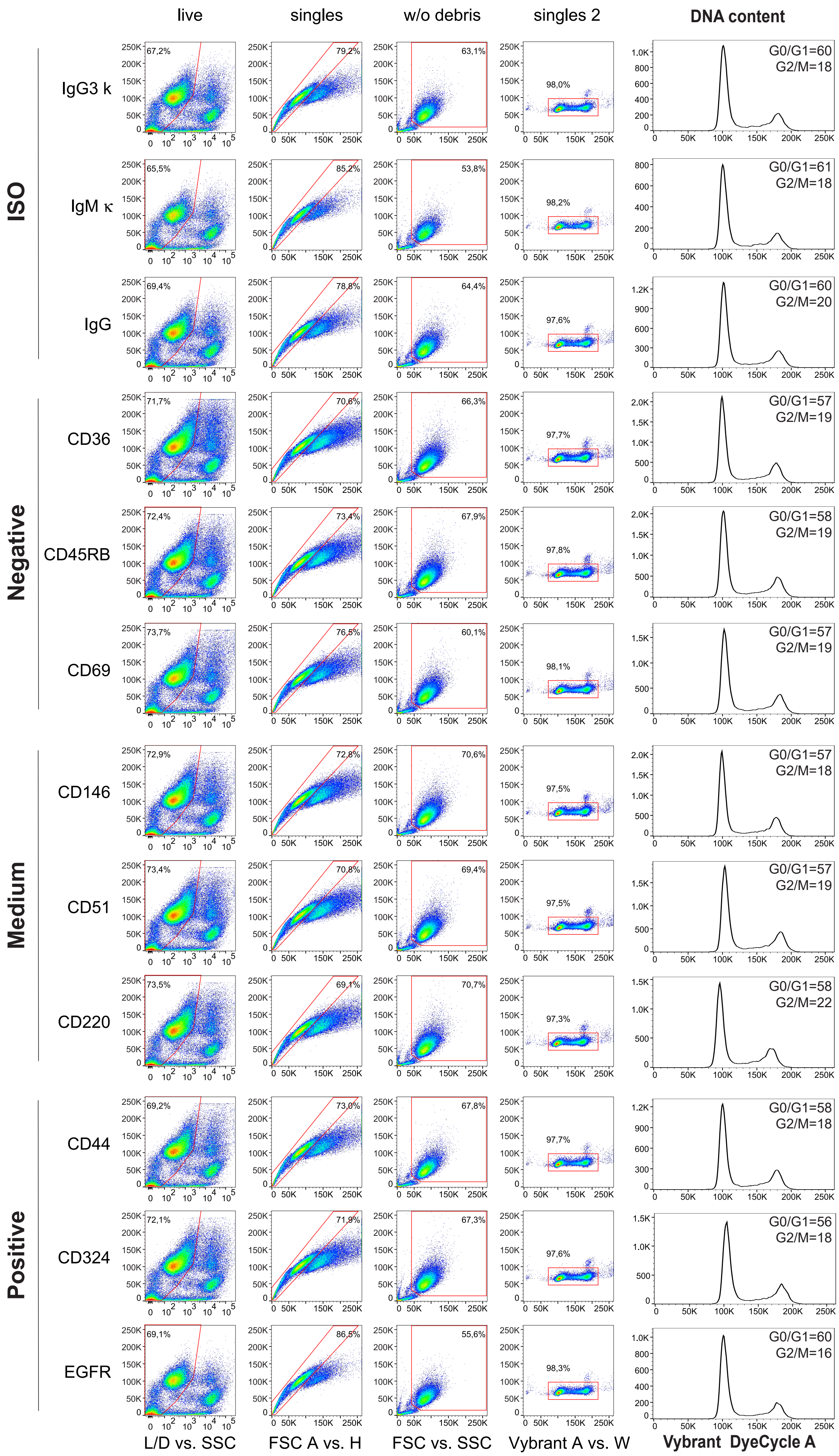

Supplementary Figure 5

Post sorting

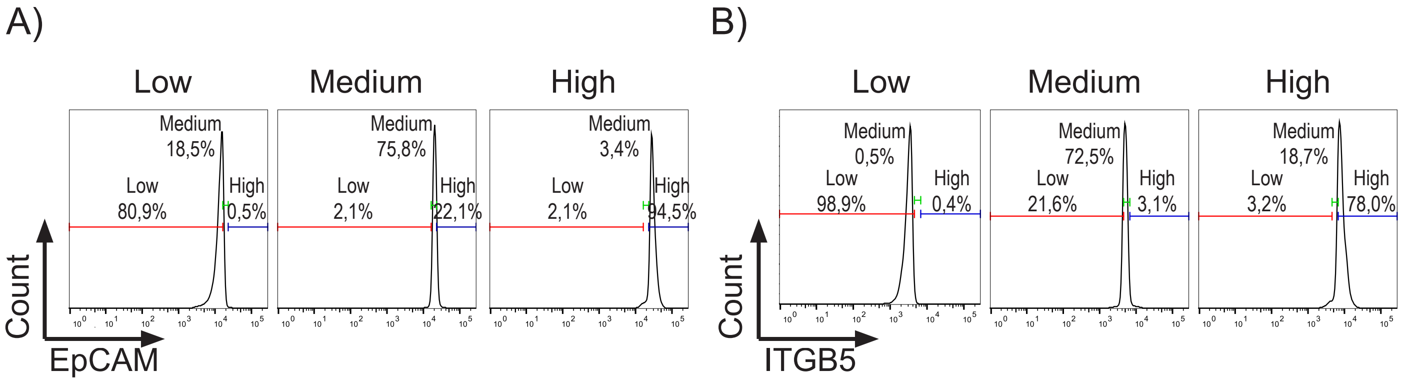

Supplementary Figure 6

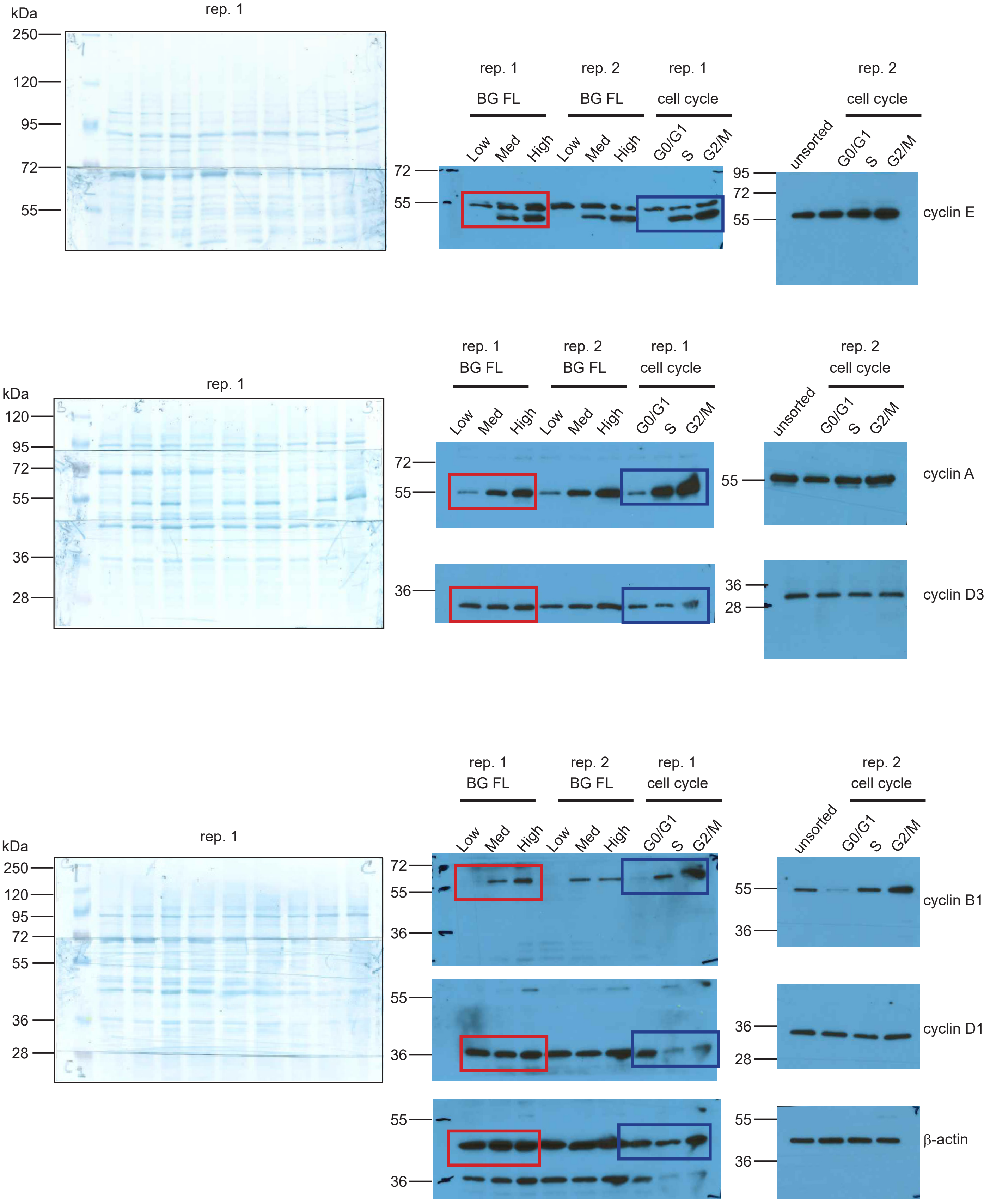

Supplementary Figure 7

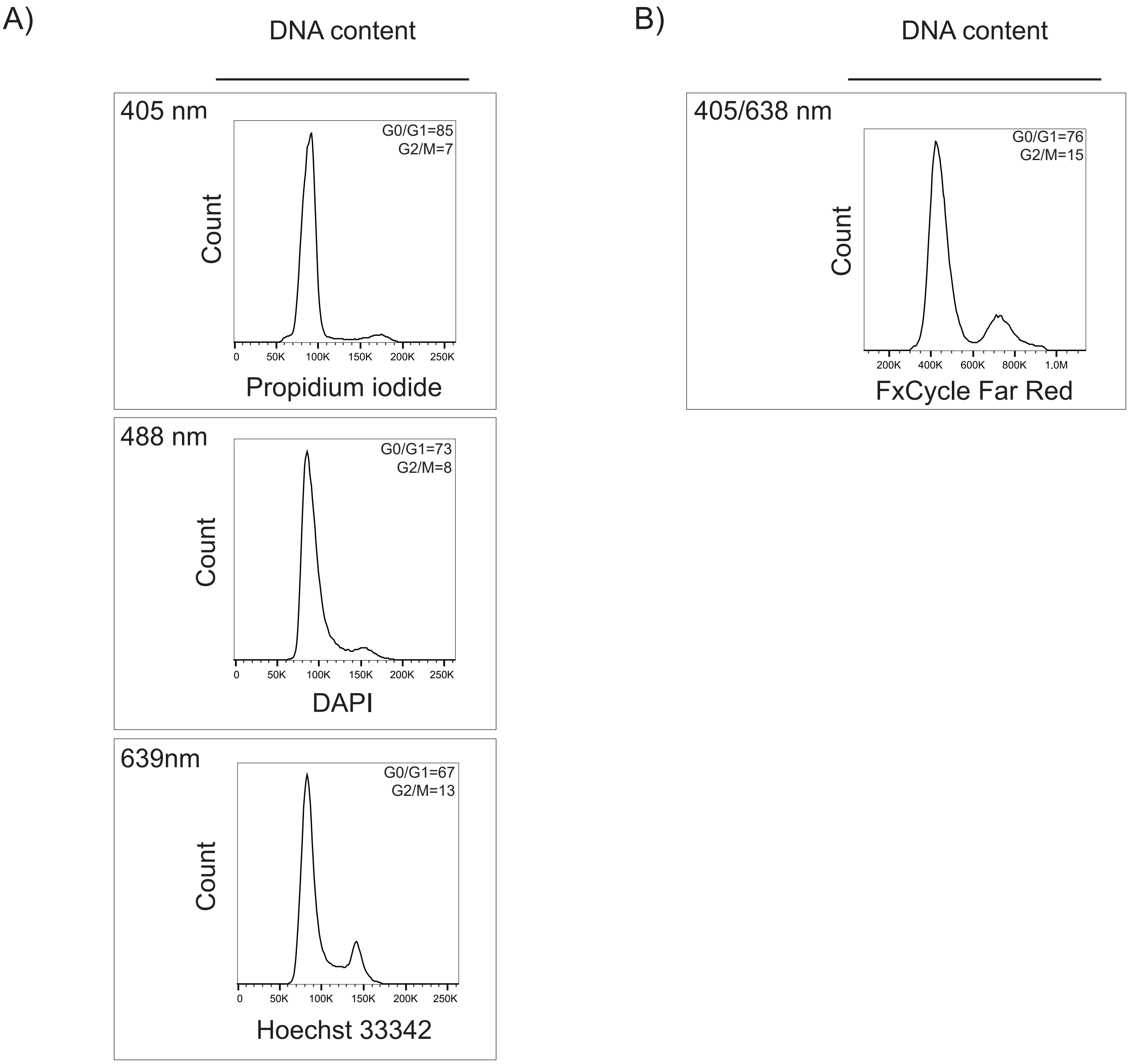

Supplementary Figure 8
