# Supplementary material for: Variability of fluorescence intensity distribution measured by flow cytometry is influenced by cell size and cell cycle progression
Source: Sci Rep. 2023 Mar 25;13:4889. doi: 10.1038/s41598-023-31990-1 (PMC10039904; doi:10.1038/s41598-023-31990-1)
Supplement: Supplementary file 3 — Supplementary Table S1. [file 41598_2023_31990_MOESM3_ESM.docx]

Supplementary table S1: Configuration of flow cytometers

| Laser (nm) | Manufacturer | Power (mW) | Detector | Dichroic Mirror // Band Pass Filter (nm) | Installation (year) | Instrument |
| --- | --- | --- | --- | --- | --- | --- |
| 405 | Power Technology | 50 | VL1 | 410LP // 450/40 | 2013 | Attune |
| 405 | Power Technology | 50 | VL2 | 500LP // 522/31 | 2013 | Attune |
| 405 | Power Technology | 50 | VL3 | 575LP // 603/48 | 2013 | Attune |
| 488 | Spectra-Physics | 20 | BL1 | Empty // 530/30 | 2013 | Attune |
| 488 | Spectra-Physics | 20 | BL2 | 555LP // 574/26 | 2013 | Attune |
| 488 | Spectra-Physics | 20 | BL3 | 620LP // 640LP | 2013 | Attune |
| 355 | Coherent Genesis | 60 | A - Trigon | 505LP // 530/30 | 2009 | FACSAria II SORP |
| 355 | Coherent Genesis | 60 | B - Trigon | Empty // 450/50 | 2009 | FACSAria II SORP |
| 405 | Coherent Cube | 100 | A - Octagon | 750LP // 780/60 | 2009 | FACSAria II SORP |
| 405 | Coherent Cube | 100 | B - Octagon | 670LP // 710/50 | 2009 | FACSAria II SORP |
| 405 | Coherent Cube | 100 | C - Octagon | 630LP // 660/20 | 2009 | FACSAria II SORP |
| 405 | Coherent Cube | 100 | D - Octagon | 595LP // 605/40 | 2009 | FACSAria II SORP |
| 405 | Coherent Cube | 100 | E - Octagon | 505LP // 525/50 | 2009 | FACSAria II SORP |
| 405 | Coherent Cube | 100 | F - Octagon | Empty // 450/50 | 2009 | FACSAria II SORP |
| 488 | Coherent Sapphire | 100 | A - Octagon | 670LP // 685/35 | 2009 | FACSAria II SORP |
| 488 | Coherent Sapphire | 100 | B - Octagon | 505LP // 525/50 | 2009 | FACSAria II SORP |
| 561 | Coherent Sapphire | 100 | A - Octagon | 750LP // 780/60 | 2013 | FACSAria II SORP |
| 561 | Coherent Sapphire | 100 | B - Octagon | 685LP // 710/50 | 2013 | FACSAria II SORP |
| 561 | Coherent Sapphire | 100 | C - Octagon | 635LP // 670/30 | 2013 | FACSAria II SORP |
| 561 | Coherent Sapphire | 100 | D - Octagon | 600LP // 610/20 | 2013 | FACSAria II SORP |
| 561 | Coherent Sapphire | 100 | E - Octagon | 570LP // 585/15 | 2013 | FACSAria II SORP |
| 640 | Coherent Cube | 40 | A - Trigon | 750LP // 780/60 | 2009 | FACSAria II SORP |
| 640 | Coherent Cube | 40 | B - Trigon | 685LP // 710/50 | 2009 | FACSAria II SORP |
| 640 | Coherent Cube | 40 | C - Trigon | Empty // 670/14 | 2009 | FACSAria II SORP |
| 488/635 | Spectra-Physics | 15 | FL1 | 560SP // 530/30 | 2008 | FACSCalibur |
| 488/635 | Spectra-Physics | 15 | FL2 | Empty // 585/42 | 2008 | FACSCalibur |
| 488/635 | Spectra-Physics | 15 | FL3 | 640LP // 670LP | 2008 | FACSCalibur |
| 488/635 | Spectra-Physics | 15 | FL4 | 640LP // 661/16 | 2008 | FACSCalibur |
| 405 |  | 40 | A - Digon | 448/45 // 448/45 | 2012 | FACSVerse |
| 405 |  | 40 | B - Digon | 500LP // 528/45 | 2012 | FACSVerse |
| 488 |  | 20 | A - Hexagon | 752LP // 783/56 | 2012 | FACSVerse |
| 488 |  | 20 | B - Hexagon | 665LP // 700/54 | 2012 | FACSVerse |
| 488 |  | 20 | D - Hexagon | 560LP // 584/42 | 2012 | FACSVerse |
| 488 |  | 20 | E - Hexagon | 507LP // 527/32 | 2012 | FACSVerse |
| 640 |  | 40 | A - Digon | 752LP // 783/56 | 2012 | FACSVerse |
| 640 |  | 40 | B - Digon | 660/10 // 660/10 | 2012 | FACSVerse |
| 405/638 |  | 60 | 32 channel PMT  PMT x 2 | 32 channel PMT (wavelength: 500-800nm)  PMT x 2 (420-440nm, 450-470nm) | 2015 | SP6800 Spectral Analyzer |
| 488 |  | 40 | 32 channel PMT | PMT (wavelength: 500-800nm) | 2015 | SP6800 Spectral Analyzer |
