# Supplementary figures and images for: Variability of fluorescence intensity distribution measured by flow cytometry is influenced by cell size and cell cycle progression
Source: Sci Rep. 2023 Mar 25;13:4889. doi: 10.1038/s41598-023-31990-1 (PMC10039904; doi:10.1038/s41598-023-31990-1)

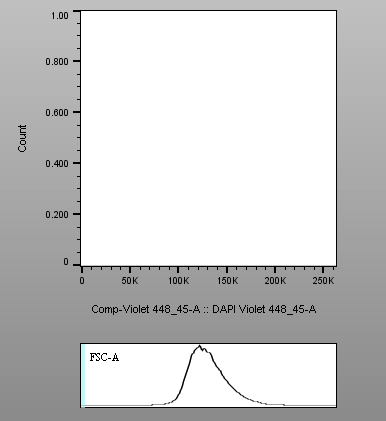

Supplement: Supplementary file 4 — Supplementary Information 1. [file 41598_2023_31990_MOESM4_ESM.gif]

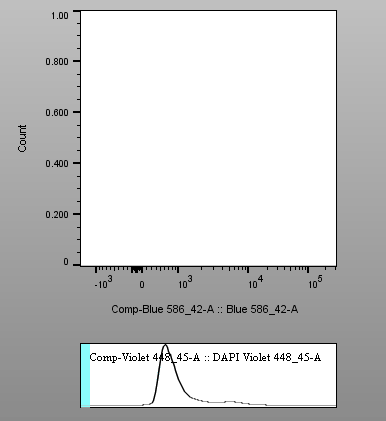

Supplement: Supplementary file 5 — Supplementary Information 2. [file 41598_2023_31990_MOESM5_ESM.gif]
